# Sexual Dysfunction in Cervical Cancer Survivors: A Scoping Review

**DOI:** 10.1089/whr.2021.0035

**Published:** 2021-12-07

**Authors:** Neha Mishra, Nilanchali Singh, Mohini Sachdeva, Prafull Ghatage

**Affiliations:** ^1^ Department of Obstetrics and Gynaecology, GIMS, Greater Noida, India.; ^2^ Department of Obstetrics and Gynaecology, All India Institute of Medical Sciences (AIIMS), New Delhi, India.; ^3^ Department of Gynaecologic Oncology, Tom Baker Cancer Centre, University of Calgary, Calgary, Alberta, Canada.

**Keywords:** sexual dysfunction, cervical cancer, survivors, vaginal dryness, dyspareunia, vaginal stenosis, libido

## Abstract

Sexual function in cervical cancer survivors declines significantly after treatments irrespective of the modality used. Only a few studies have looked at their psychosexual needs, perception, and acceptance of psychosexual support. This review summarizes findings of current qualitative as well as quantitative studies to understand the plight of cervical cancer survivors regarding sexual dysfunction and the management issues. The effect of gynecologic cancers on sexuality depends on multiple factors such as psychosexual factors, biologic factors, and age. Younger patients have poorer outcomes with a more pronounced impact on sexual well-being. Radicality of surgery has direct correlation with sexual dysfunction. Low or no sexual interest, lack of lubrication, dyspareunia, and reduced vaginal caliber are frequently found. For too long, researchers have focused on defining the prevalence and types of sexual problems after various cancer treatments. The area that continues to be neglected is the evaluation of effective interventions to prevent or treat cancer-related sexual dysfunction. In particular, mental health and medical specialists need to collaborate to create cost-effective treatment programs. Collaborative intervention with gynecologists, sexologists, radiotherapists, and nursing staff would be beneficial to optimize the sexual wellness of cancer survivors and their spouses.

## Introduction

Cervical cancer has improved prognosis over the years, due to effective screening and multiple treatment modalities, with 5-year survival rate of 60%.^1^ The number of women surviving cervical cancer is on the increase, but with substandard quality of life (QOL). The quality of life of cervical cancer survivors is poorer not only when compared with the healthy age-matched general population but also when compared with other gynecological cancer survivors.^2^

The World Health Organization (WHO) states that sexuality is one of the important indicators of quality of life with its linkage to thoughts, feelings, actions, social integration, and therefore, physical and mental health and well-being.^3^ The cervical cancer survivors suffer sexual dysfunction at multiple levels with hindrance at one or more phases of the sexual response cycle such as libido, arousal, orgasm, and resolution.^4^

Sexual dysfunction is prevalent in these women, and vaginal dryness, dyspareunia (superficial and deep) due to vaginal stenosis, and loss of libido are often experienced by them. The pain and psychological distress render them prone to poorer sexual function.^5–13^ In general, sexual function in cervical cancer survivors declines significantly after treatments irrespective of the modality used.^14–20^

Only a few studies have looked at their psychosexual needs, perception, and acceptance of psychosexual support. Most of the studies have focused on the QOL in general in all the gynecological cancer survivors. However, we have limited ourselves to sexual dysfunction experienced by cervical cancer survivors only to elicit the importance of this issue and to present a detailed description of the topic. This review summarizes findings of the current qualitative as well as quantitative studies to understand the plight of cervical cancer survivors regarding sexual dysfunction, and with special focus on the management issues.

## Methodology

A review protocol was developed. The search was conducted in the PubMed, Embase, and Google Scholar databases, using the keywords “cervical cancer” or “cervical neoplasms” and “sexual dysfunction.” The search included original articles, review articles, qualitative studies, and ongoing trials published from 1999 onward in English that evaluated sexual function in women treated for cervical cancer. A total of 306 articles were screened and 112 were included in the review. The studies evaluating sexual dysfunction in other gynecological cancers (endometrial, ovarian, vaginal, and vulvar), including cervical cancers, were also included for reference.

This methodology was adopted as we wanted this to be a comprehensive review and did not want to be overwhelmed with data.

To select the studies, an initial reading of the retrieved abstracts was carried out, excluding articles with a sample that did not contain women treated for cervical cancer. The inclusion criteria of articles were articles published after 1999, in English, availability of full text and discussing sexual dysfunction in cervical cancer survivors exclusively, or including cervical cancer as a subgroup among other gynecological cancers. The articles not fulfilling the inclusion criteria were excluded. The exception has been made in therapies section where articles discussing pelvic floor therapy and arousal devices do not include cervical cancer patients specifically.

**FIG. 1.**
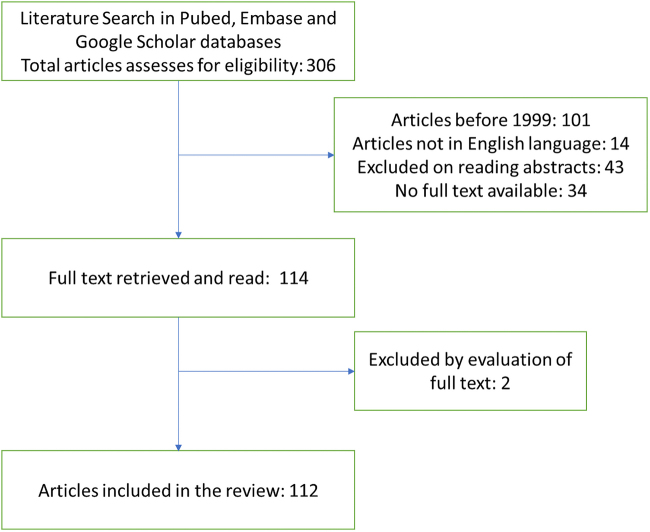
Selection of articles.

However, it was necessary to include them in the review to open future avenues of research. Subsequently, the articles were accessed in full, and from these articles the following information was extracted for the compilation of the results: characteristics of the sample under study, comparison groups, instruments and methods used, main findings, and conclusions. The data were summed up as a scoping review. Refer to Figure 1.

## Magnitude of Problem

The effect of gynecologic cancers on sexuality depends on multiple factors such as psychosexual factors, biologic factors, and age.^21^ Younger patients have poorer outcomes with a more pronounced impact on sexual well-being. Radicality of surgery has direct correlation with sexual dysfunction.^22,23^ The studies reported the sexual dysfunction in women treated for cervical cancer with varying prevalence: loss of sexual interest in 26%–85%, diminished lubrication in 27%–35%, dyspareunia in 26%–55%, dissatisfaction with sexual life in 30%–37%, narrow/short/dry vagina in 32%–50%, reduced intercourse in 45%, and orgasmic dysfunction in 20% of women treated with either radiotherapy or radical hysterectomy with pelvic lymphadenectomy.

The prospective studies assessing changes in sexual function longitudinally observed that persistent sexual dysfunction and adverse vaginal changes were reported 2 years after radiotherapy, with only insignificant improvements over time. The patients who underwent radical hysterectomy experienced severe orgasmic problems and uncomfortable sexual intercourse due to the compromised vaginal size during the first 6 months after surgery, with lack of sexual interest and diminished lubrication persisting up to 2 years.^8,11,13,24^

Greimel et al. did a study to investigate the long-term treatment side effects on the quality of life and sexual functioning of cervical cancer survivors undergoing different treatment modalities. One hundred twenty-one patients (63 surgery, 38 surgery/chemotherapy [CT], and 20 surgery/radiotherapy [RT]) were recruited.^25^ A total of 43.3% were not sexually active and the reported reasons were nonavailability of partners (24.8%), lack of interest (23.1%) or partner not interested (9.1%), physical issues (13.2%), partner has some problems (12.4%), fatigue (9.9%), partner fatigued (8.3%), and other reasons (5%). Regarding “sexual frequency,” patients in the surgery/RT group reported a significantly lower sexual activity rate compared with women in the surgery group or women in the surgery/CT group.^19^

Another study reported that 58.7% of women attributed cervical cancer/treatment for interposing with their sexual lives. A total of 73.91% of cervical cancer survivors considered sexual activity to be an important aspect in their lives. Only 15 out of 46 (32.6%) had sexual intercourse 3 months after treatment completion. Among these 15 women, two-thirds had sexual dysfunction as per low Female Sexual Function Index (FSFI) scores, notably in the domains of pain and satisfaction. Radiotherapy affected the lubrication and pain domains the most.^26^

## Pathophysiology of Sexual Dysfunction in Cervical Cancer Survivors

### Sexual function after cancer diagnosis

Aerts et al. reported that the cervical cancer patients experienced significantly more entry dyspareunia and reduced orgasm and sexual arousal compared with healthy controls. They found that the diagnosis of cervical cancer itself hampers the quality of life of the patients. They were found to be more depressed compared with their healthy counterparts. However, the quality of relationship with the partners was significantly superior in cervical cancer patients, which deteriorated at 6 months after surgery.

This change in relational functioning could be attributed to the stress and dilemmas associated with the deciding treatment plan and other family-related issues. They reported a notable finding that there was not much discrepancy in sexual function in cervical cancer patients pre- and postsurgery. This might be explained by the changed outlook regarding life and modification of internal standards after cervical cancer diagnosis.^27^

Another study reported women diagnosed with earlier stages of cervical cancer perform better regarding sexual activity compared with women diagnosed with advanced stages. Sexual activity was comparable among women diagnosed with cervical cancer and the general population. Sexual dysfunction was less likely in Caucasians compared with other races and more in women with an annual income less than 59,999 Euros. However, this study did not find any significant difference in sexual activity in women diagnosed with cancer at an earlier age (≤38 years).^28^

### Sexual dysfunction after surgery

Medical or surgical menopause leads to sudden lowering in levels of estrogen, testosterone, and progesterone in cervical cancer survivors. This endocrinal change results in vaginal thinning, vulvovaginal atrophy, clitoral atrophy, decreased vaginal elasticity, vaginal dryness, and onset of dyspareunia.^29–36^ Acute symptoms are dyspareunia, orgasmic difficulty, lack of sexual satisfaction, and distress by a reduced vaginal caliber during intercourse. Chronic symptoms include reduced libido and vaginal lubrication. Radical hysterectomy can reduce vaginal blood flow during arousal. Due to disruption of hypogastric and splanchnic nerve plexuses during surgery, bladder and bowel function gets affected causing urinary retention, urgency, and constipation indirectly affecting sexual function.^37^

A review of the effects of surgery on sexual function found that there are different schools of thought regarding radical hysterectomy and its impact on sexual dysfunction. Some studies concluded that sexual function scores declined initially, but they were not different from controls on long-term follow-up. The rationale behind this conclusion was that vaginal/sexual function undergoes a sharp recovery after a few years, although it seemed to be compromised during the first months following radical hysterectomy.

Other studies concluded that patients who underwent radical hysterectomy had severe sexual dysfunction. Although scores improved over time, it never reached those of their healthy counterparts. Fertility sparing/laparoscopic surgeries have outcomes comparable with open surgeries regarding sexual dysfunction (Table 1).^14,38–41^

**Table 1.** Effect of Surgery on Sexual Function

S. No.	Study	Aim	Conclusions
1.	Frumovitz et al.^14^	Comparison of QOL and sexual health in cervical cancer survivors treated with radical hysterectomy with lymph node dissection to that with radiotherapy.	• Radiation patients had worse sexual functioning. • No difference between radical hysterectomy group and controls regarding sexual functioning.
2.	Chan et al.^38^	Comparison of sexual dysfunction in women with cervical cancer, treated with FSS or no FSS.	No significant difference in sexual functions or sexual QOL mean scores in women who underwent FSS (cold-knife cone or trachelectomy), after adjusting for age and menopausal status.
3.	Xiao et al.^39^	Cervical cancer patients, who were sexually active survivors and had undergone type II/III radical hysterectomy and/or lymphadenectomy, laparoscopically or by laparotomy, were evaluated.	The future of quality of life and sexual health of patients was not found to be related to the surgical approach chosen.
4.	Fleming et al.^40^	Prospective study with early-stage cervical cancer before undergoing radical trachelectomy and postoperatively at 6 weeks, 6 months, 1 year, and annually thereafter for 4 years.	The scores for arousal (*p* < 0.001), lubrication (*p* < 0.0011), orgasm (*p* = 0.006), pain (*p* = 0.01), satisfaction (*p* = 0.03), and total score (*p* = 0.004) showed a significant decline at 6 weeks and then returned to baseline levels by 6 months. The assessment for desire showed no significant change in score from baseline throughout the follow-up period. In a subset analysis, there were no differences in QOL scores based on an open versus minimally invasive surgical approach.
5.	Plotti et al.^41^	Retrospective study protocol in cervical cancer patients having complete response to treatment (type III radical hysterectomy), for locally advanced cervical cancer with at least 36 months of follow-up.	Concerning sexual activity, data indicated a good level of sexual enjoyment with a slight worsening of sexual activity.

FSS, fertility sparing surgery.

### Sexual dysfunction after radiotherapy/chemotherapy

Radiation works by a random process that damages tumor cells. Similar damage may occur to normal cells adjacent and surrounding the tumor. The cervix, body of the uterus, vagina, bladder, and rectum have similar structures—epithelium, stroma, muscle layer, and blood vessels. Acute radiation damage to the mucosa and submucosa leads to acute tissue edema and mucosal and submucosal inflammation. The rapid rate of cell turnover in the vaginal and vulval epithelium makes them vulnerable to the effects of radiation.

Acute radiation symptoms include vaginal erythema, moist desquamation, and mucositis. Chronic symptoms include thinned out vaginal, adhesions, atrophy, and fibrosis. This is followed by decreased vaginal elasticity, narrowing, shortening, and ultimately, total vaginal length.^42^

The combination of surgery and radiation is associated with more vaginal shortening compared with radiation alone. Chemotherapy in women also results in sexual dysfunction and loss of sexual interest due to poor self-esteem as a consequence of postchemotherapy changes such as hair loss and negative body image. However, the effect of radiotherapy was very profound compared with chemotherapy.^43,44^

Radiotherapy can destroy the surrounding healthy tissues and organs too, which are adjacent to the tumor. This is related to damage to the stroma and vascular endothelium leading to sexual, fecal, and urinary disorders.^45–47^ The effect of radiotherapy on sexual function is worse than that of radical hysterectomy and pelvic lymphadenectomy. Radiotherapy creates persistent vaginal atrophy even years after treatment. The newer studies suggest that new techniques in radiotherapy may result in improved sexual functions in cervical cancer survivors (Table 2).^48–53^

**Table 2.** Effect of Radiotherapy on Sexual Function

S. No.	Study	Aims	Conclusions
1.	Korfage et al.^49^	Cervical cancer-specific HRQoL and anxiety were assessed and compared with a reference population.	RT was associated with an increased frequency of treatment-related side effects even after 2–10 years. More sexual worry and worse body image were seen in 2–5-year than 6–10-year survivors.
2.	Jensen and Froeding^51^	Studies on gynecological, urological, and gastrointestinal cancers were included from 2010 to 2014 where FSD at least constituted a secondary outcome.	Pelvic radiotherapy has a continuous deteriorating effect on the mucosa of vagina affecting sexual function negatively in female cancer patients. They also expected modern radiotherapy to cause less vaginal morbidity.
3.	Gargiulo et al.^52^	LACC in remission after treatment with NACT+RS (*n* = 34) or CT/RT were interviewed to compare long-term toxicity and QOL.	NACT+RS group had worse sexual life perception.
4.	Daga et al.^104^	Survivors of locally advanced cervical cancer older than 48 years (stages IIB–IVA) who completed concurrent CT/RT along with intracavitary brachytherapy at least 2 years prior were recruited.	The mean scores were 2.3, 2 and 2.1 for sexual desire, arousal, and dyspareunia, respectively. The total mean score was 11.84 (range: 3.2–19.5) with a cutoff of 26.55. All survivors exhibited female sexual arousal disorder.
5	Rahman et al.^48^	Assessment of the QOL in women suffering from cancer of cervix before and after the treatment.	No statistically significant difference in QOL of patients treated with either surgery or radiotherapy Sample size too small to comment on differences.
6	Kumbhaj et al.^53^	Comparison of QOL and sexual functions in cervical cancer survivors treated with surgery to that with radiotherapy.	Cervical cancer survivors treated with radiotherapy had worse sexual functioning than did those treated with radical hysterectomy and lymph node dissection.

CT, chemotherapy; FSD, female sexual dysfunction; HRQoL, health related quality of life; LACC, laparoscopic approach to cervical cancer; NACT, neoadjuvant chemotherapy; QOL, quality of life; RS, radical surgery; RT, radiotherapy.

## Methods of Assessment of Sexual Function

Cervical cancer survivors should be enquired about their sexuality at frequent intervals. Their sexuality should be assessed before cancer, at diagnosis, and after treatment along with their marital relationship.^54^ Complete history, including past, medical, surgical, sexual, personal, and treatment history, should be taken with particular attention to medications affecting sexual function. Thorough examination of the pelvis, and external and internal genitalia to see edema, scarring, stenosis, infection, ulcers, pelvic muscle strength, and so on should be done. Q-tip test can be used for localizing vulvar pain.^55^

Various scales for assessment of sexual dysfunction have been used objectively. Quality-of-life questionnaires are varied as per their content and coverage. Some are general questionnaires applicable to any population with any health condition (generic form of questionnaire), whereas others are focused on specific diseases such as cervical cancer (disease-based specific questionnaires).

The questionnaires such as Functional Assessment of Cancer Therapy-Cervix (FACT-Cx) assess general well-being of cervical cancer patients. The determination of quality of life in cervical cancer survivors is imperative to provide holistic treatment to cervical cancer survivors.^56^ In addition, dimension-specific questionnaires focusing on sexual dysfunction, depression, anxiety, or other adverse effects are also used. We are discussing some of the available instruments focused on sexual dysfunction below.

### The European Organization for Research and Treatment of Cancer Quality-of-Life Questionnaire Cervix Module 24

The EORTC Cervical Cancer Module 24 (QLQ-CX24) was developed to supplement the EORTC QLQ-C30 core questionnaire to assess the impact of common cervical cancer treatment quality of life. This scale includes 24 items consisting of 3 multi-item scales (symptom experience, body image, and sexual and/or vaginal functioning) and 5 single-item scales. It is a disease-specific and treatment-specific measurement tool. It has been validated in patients with cervical cancer with internal consistency (Cronbach's coefficients range, 0.72–0.86 in multi-item scales such as experience 0.72, body image 0.86, sexual/vaginal functioning 0.87) and fewer scaling errors.^57^

### Female Sexual Function Index

The FSFI is a multidimensional survey containing 19 items that caters to 5 domains of sexual health, including sexual desire, arousal (both subjective and physiological), lubrication, orgasm, satisfaction, and pain. Higher scores indicate better sexual functioning. The FSFI is the most commonly used instrument to measure sexual dysfunction with initial excellent reliability for total score (Cronbach's α coefficient, 0.97) and subscales (Cronbach's α coefficient, 0.89–0.96).^58^ FSFI is widely used in the gynecologic cancer literature.

### Leiden Questionnaire

The Leiden Questionnaire was designed to measure sexual function and vaginal changes for patients with gynecological cancer and was proven to be reliable (Cronbach's α 0.73–0.80, 66 patients treated for cervical cancers included). Leiden questionnaire is a questionnaire comprising 14 items: physical (3 items) and sexual (11 items) symptoms. Only the sexual part is validated. It is a dimension-specific questionnaire.^59^

### Sexual Function-Vaginal Changes Questionnaire

These are dimension-specific measurements concentrated on sexual function. The Sexual Function-Vaginal Changes Questionnaire has good patient/observer agreement. This instrument containing 20 core items focuses on quantifying sexual inclinations, orgasm, lubrication, painful sexual intercourse, vaginal length, intimacy, sexual activity, sexual issues pertaining to partner, sexual satisfaction, and body image. The Sexual Function-Vaginal Changes Questionnaire was tested in 75 patients with gynecological cancer, and the level of patient/observer agreement was high (0.84). (The Cronbach's α for the total score was 0.94 and subscales ranged from 0.85 for the satisfaction domain to 0.94 for the lubrication domain.)^60^

### Cancer Rehabilitation Evaluation System-Shorter Format

It is a disease-specific questionnaire with the number of items between 38 and 49. The Cancer Rehabilitation Evaluation System-Shorter Format (CARES-SF) is highly related to the CARES, with good retest reliability, concurrent validity, and acceptable internal consistency of summary scales.^61^

### Sexual Activity Questionnaire

This questionnaire has 21 items, dimension specific with a 3-factor structure of the Sexual Activity Questionnaire (SAQ)-functional scale: habit, pleasure, and discomfort from sexual intercourse. The internal consistency of the SAQ-functional scale showed a Cronbach's coefficient alpha of 0.86.^62^

### Functional Assessment of Cancer Therapy-Cervix

The FACT-Cx is the modification of functional assessment of cancer therapy-general (FACT-G) with an addition of cervix subscale. The FACT-G measures the quality of life of cancer patients. This instrument of 27 questions has 4 domains: physical, social/family, emotional, and functional well-being. The cervix subscale comprises 15 questions for patients with cervical cancer. Scores range from 0 to 108 for the FACT-G, and 0 to 60 on the cervix subscale.^63,64^

### Menopause-Specific Life Questionnaire

Menopause-Specific Life Questionnaire (MEN-QOL) is a questionnaire pertaining to disease. MEN-QOL encompasses 29 items in the form of Likert scale arrangement. Every item assesses the impact of menopausal symptoms by catering to 1 of 4 domains: vasomotor (items 1–3), psychosocial (items 4–10), physical (items 11–26), and sexual (items 27–29).^65^

### World Health Organization Quality of Life Assessment-Bref

This established survey instrument can be used as a proxy to assess sexual quality of life using three questions pertaining to social health. This questionnaire contains a domain for social health, including questions regarding personal life, social support, and sexual activity.^66^

### The Satisfaction with Life Scale

In this questionnaire, Likert-type scale is used (“very dissatisfied,” “dissatisfied,” “neither satisfied nor dissatisfied,” “satisfied,” and “very satisfied”) for questions related to sexual life. The Satisfaction with Life Scale is known for its beneficial psychometric properties, including high internal consistency and high temporal reliability.^67^

### The Female Sexual Distress Scale-Revised

The Female Sexual Distress Scale-Revised (FSDS-R) has shown excellent discriminant validity, high test/retest reliability, and a high degree of internal consistency in measuring personal distress pertaining to sexuality in women with hypoactive sexual desire dysfunction. FSDS-R item 13 alone also demonstrated reasonable discriminant validity and test/retest reliability. Higher scores indicate more sexual dissatisfaction.^68^

### The Golombok Rust Inventory of Sexual Satisfaction

This contains 28 items and covers the most frequently occurring sexual complaints of women. Seven subscale scores can be derived: anorgasmia, vaginismus, reduced frequency of sexual contact, sexual noncommunication, dissatisfaction, nonsensuality, and avoidance of sex. Higher scores indicate more problems.^69,70^

## Changes in Pattern of Sexual Dysfunction with Respect to Time

When we investigated changes in pattern of sexual dysfunction over a period of time in cervical cancer survivors, we found that quality of life and sexual dysfunction declined in the immediate postoperative period until 6–12 months.^48,71^ After that, sexual function improved on long-term follow-up, but it never reached that of their healthy counterparts.^10,49^

## Impact of Partners' Involvement on Sexual Function

It was found that emotional support from the partners positively affects sexual QOL reinforcing the importance of this support and of counseling by health professionals, for the women and their partners, about the disease and sexuality.^72^ However, both patients and partners feel inhibited to initiate sexual intercourse and enjoying sexual life due to fear, communication difficulties, pain, guilt, grief, fear of reinfection, and lack of desire. These emotional elements endanger the coherence of their relationships and leads to verbal abuse and domestic conflict. Some women force themselves to be sexual active out of fear of not fulfilling their responsibility.^73–75^

Hence, the relationship between cervical cancer survivors and their partners is complex and significant support may be required from their health care providers. Sexual abuse by the spouse, anger and stress due to refusal of sex by the partner, and an increased divorce rate have been reported in cervical cancer survivors.^31,76,77^ Most patients believe that partners should be provided with information and education on how to support their spouse in case of sexual concerns and also address a possible need for support on their side. This psychosexual support should therefore not only encompass the physical aspect but also consider the relationship and partner's perspective.^78^

## Factors Affecting Sexual Function in Cervical Cancer Patients

Etiology for sexual dysfunction in cervical cancer is multifactorial.^56,71,73,78,79^ There are many factors that play along to decide the sexual QOL of cervical cancer survivors. As per Zhou et al., quality of life including sexual functioning was determined by multiple social factors such as availability of health insurance and white collar job, apart from clinical factors such as age, treatment-associated complications, and sleep disturbances. They reported that nonpossession of health insurance led to delay in seeking medical care resulting in upstaging of disease, which hampered sexual function significantly. The women with occupation exhibited better sexual functioning probably due to better educational status and lesser number of women receiving radiotherapy.^56^

The role of financial instability in determining quality of life and sexual activity has been discussed by other studies as well.^71^

Another study highlighted the importance of nursing care intervention, duration of marriage, knowledge of patients, as well as education and occupation of the partners in improving the symptoms of sexual dysfunction. This study reported that the nursing care intervention is the single-most important determinant with other factors as contributory in improving sexual function.^73^ The availability of psychoeducational website and online support groups was welcomed by cervical cancer survivors for optimum sexual functioning. However, they preferred face-to-face consultation with their health care provider in case of complex issues.^78^ Table 3 shows factors determining sexual function in cervical cancer survivors.

**Table 3.** Factors Determining Sexual Function in Cervical Cancer Survivors

S. No.	Study	Factor
1.	Zhou et al.^56^	Age
2.	Zhou et al.^56^	Radiotherapy
3.	Zhou et al.^56^	Type of surgery
4.	Zhou et al.^56^	Sleep disorders
5.	Zhou et al.^56^	Health insurance availability
6.	Zhou et al.^56^	Occupation
7.	Vermeer et al.^78^	Availability of psychoeducational website and online support groups
8.	Afiyanti et al.^73^	Education
9.	Afiyanti et al.^73^	Physical and relaxation exercises
10.	Afiyanti et al.^73^	Availability of counseling and social support groups
11	Sabulei and Maree^71^	Financial difficulties during time of treatment

## Impact of Role of Medical Care Providers on Sexual Dysfunction

The medical care providers could be the game changers in improving sexual function in cervical cancer survivors. Their role is listed in Table 4.^73,78,80–83^ Many studies have highlighted the importance of communication and counseling between medical care providers and patients. The pressures on gynecologic oncology surgeons are gigantic and discussions about sexual health can be challenging. By simply asking about sexual health, providers can make the patient comfortable to discuss concerns and remove doubts about sexuality postcancer treatment.^83,84^

**Table 4.** Role of Medical Care Providers

S. No.	Study	Role of medical care providers
1.	Afiyanti et al.^73^	Nursing care intervention on sexuality through educational counseling.
2.	Vermeer et al.^78^	Psychosexual support should go one step ahead of physical sexual functioning and focus on aspects such as sexual distress, relationship satisfaction, and partner perspective.
3.	Vermeer et al.^78^	Gynecologists/physicians to offer more practical information about sexuality and relationship consequences. Help from sexologists should be sought.
4.	Schover et al.^80^	Health care providers should inform and help with the sexual consequences of cancer treatment.
5.	Falk et al.^81^	The patient should be asked open-ended questions. There should be no judgmental attitude regarding sexual orientation or sexual practices.
6.	Huffman et al.^82^	Doctors/nurses must understand, evaluate, and treat sexual health issues encountered during treatment and survivorship.
7.	Shankar et al.^83^	Physicians should consider referral of patients to an expert if need arises (complex or intractable issues).

A well-organized system of asking, assessing, and then reaching a plan of management should be incorporated into a busy clinical practice. The patients affected by cervical carcinoma are often of young age having a life expectancy of 25–30 years on an average. Therefore, the quality of life and sexual functioning are crucial for health care providers.^85^

## Therapies to Improve Sexual Dysfunction

While reviewing the studies on treatment modalities for sexual dysfunction, we found that there were only few studies directed specifically for cervical cancer survivors. We have included studies on postmenopausal women as well as other gynecological cancers. Table 5 depicts the various treatment options.

**Table 5.** Treatment Modalities for Common Sexual Problems Experienced by Cervical Cancer Survivors

S. No.	Diagnosis	Symptoms	Treatment strategies
1.	Female sexual interest/arousal disorder	Physical symptoms	Treat the cause, pelvic floor therapy
		No physical cause	Counseling and specialist consultation, psychoeducational intervention
		Associated with medical conditions	Treat and evaluate again
2.	Female orgasmic disorder	Difficulty in experiencing orgasm	Treat cause
			Vibrators
			EROS-CTD
			Eroticas magazines/visuals
			Foreplay
3.	Genitopelvic pain penetration disorder	Dryness of vagina	Lubricants and moisturizers
			Hormonal therapy
		Vaginal stenosis	Lubricants and moisturizers
			Dilators
			Pelvic floor therapy

EROS-CTD, EROS clitoral therapy device.

### Pelvic floor physical therapy

It has been shown that almost every modality of cervical cancer treatment leads to pelvic floor symptoms, and hence, timely referral to pelvic floor muscle training to individuals is recommended.^86^

Pelvic floor physical therapy includes massages, pelvic floor strengthening exercises, pelvic floor relaxation exercises, intravaginal physiotherapeutic exercises, and so on. These exercises increase flexibility and improve tissue blood circulation and tensile strength in paravaginal tissues. Almost 40% of cervical cancer patients are diagnosed before the age 45 and it is being shown that young women having optimum strength in pelvic floor muscles exhibited better sexual function, especially in arousal and orgasm domains.^87,88^

Although no studies on cervical cancer survivors exist, it could be considered an option for cervical cancer survivors based on the experience of a previous study for the effectiveness of pelvic floor physical therapy in treating the pain of provoked vestibulodynia, as well as some of the sexual and cognitive correlates of provoked vestibulodynia.^89^

### Arousal devices

FDA approved the EROS-Clitoral Therapy Device (EROS-CTD) to improve female sexual function. EROS-CTD creates orgasm by gentle suction over the clitoris to increase blood flow and sensation and specifically improves problems linked to arousal and lubrication, and achieving orgasm. CTD should be used at least thrice per week to gain the maximum relief. This device had shown a potential role in patients with surgical menopause and thus could be considered for cervical cancer patients.^79^

### Nonhormonal vaginal lubricants and moisturizers

The American Cancer Society endorses the use of vaginal lubricants by patients and partners before and during sexual activity to improve sexual activity. However, the risk of candida infections may increase in some women.^90^ A study by Herbenick et al. on the general female population (2453 women) endorsed that water-based lubricants were associated with less genital symptoms compared with silicone-based lubricants. In addition, the use of a water-based or silicone-based lubricant was linked with higher sexual pleasure and satisfaction for solo sex and also compared with no lubricant.^91^

### Vaginal moisturizers and estrogen creams

The American Cancer Society suggests that the vaginal moisturizers, although not used for sexual activity usually, should be applied daily during the night for enhanced absorption and for maintaining pH and health of the vagina, which could indirectly lead to improvement in sexual function.^90^ The vaginal moisturizers popularized as bioadhesives have been reported as a safe and efficacious option in addition to estrogen vaginal cream. Both of the treatment modalities exhibit significant increase in moisture, texture, fluidity, and elasticity of vagina with a return of the premenopausal pH state.^92^

The cervical cancer survivors become menopausal either as a result of treatment or due to aging. They face acute onset of menopausal symptoms. Estrogen therapy could be considered in them.^93–95^ In a recent retrospective study by Chambers et al. on gynecological cancer survivors including 62 cervical cancer survivors (58.1% and 40.3% diagnosed with squamous cell carcinomas [SCC] and adenocarcinomas [AC], respectively) using various vaginal estrogen preparations for genitourinary syndrome after menopause, they reported that the recurrence rate was 9.7%, low compared with the recurrence rate of 30% reported by other studies.^96–99^

The use of topical estrogen has been associated with slightly less vaginal bleeding, significantly less dyspareunia, and improved vaginal health and caliber in the interventional arm compared with the control arm in a randomized study in radiation-induced vaginitis in cervical cancer patients.^100^

### Hormone replacement therapy

Faubion et al. concluded that hormonal therapy is not contraindicated in cervical, vulvar, and vaginal cancers as they are linked exclusively to HPV and not hormones as 4/5th of them are SCC. Also, they did not get any evidence regarding hormonal therapy increasing risk of carcinoma or reduced disease-free survival, as well as in AC.^101^ Similarly, Ploch studied the use of hormone replacement therapy (HRT) in cervical cancer survivors in patients younger than 45 years. After treatment, they administered HRT in 80 patients, while the remaining 40 were used as controls. In 80 patients receiving HRT- 40 received Trisequens (Novo)--(group T), and 40 with sequentially Dienestrol and Chlormadinon (Polfa)--(group D-Ch). Overall, no significant difference in recurrence rate or survival was noted between the groups.^97^

Del Carmen and Rice emphasized about the safety of HRT in cervical cancer survivors and suggested using the estrogen/progesterone combination (intact uterus) in case any estrogen responsive endometrium survives after radiotherapy.^102^

### Selective estrogen receptor modulator

Ospemifene is a third-generation selective estrogen receptor modulator approved for dyspareunia and vulvovaginal dryness by the FDA in 2013 and the European Medicines Agency in 2019.

De Rosa et al. studied the effect of ospemifene on early-stage (I/II) young ccs experiencing vulvovaginal atrophy symptoms. They found that ospemifene improved the vaginal health index significantly at 6 months of follow-up compared with baseline. Sexual function also improved along with betterment of quality of life.^103^ It may therefore be an option for women who are reluctant to use vaginal medications and/or lubricants.

### Vaginal dilator

Daga et al. strongly recommended either the use of a vaginal dilator or frequent sexual intercourse after completion of radiotherapy for cervical cancer survivors to maintain a healthy vaginal canal.^104^ A systematic review by Miles and Johnson concluded that there is no concrete evidence that routine regular vaginal dilation during RT treatment prevents stenosis or improves quality of life.^50^

### Psychoeducational interventions

It is being known that sexual dysfunction experienced by cervical cancer patients has a multifaceted etiology. The problem is not merely at the physical level. It involves various psychological issues such as mood swings, depression, loss of self-confidence, feeling of being infertile, and lack of intimate behavior with the partner.^100^ Hence to obtain optimum sexual functioning, addressing these issues is equally important. Robinson et al. reported that the psychoeducational intervention significantly improved compliance to vaginal dilator therapy and alleviated the doubts regarding sexual activity in women with stage I/II cervical/endometrial carcinoma compared with the control arm receiving written leaflets with a short explanation.^105^

A randomized-controlled trial evaluated the benefits of providing consultation regarding the patients in the interventional arm before surgery *via* a gynecologic oncology specialist nurse and standard care in the control arm. Sexual satisfaction was significantly more in the interventional arm.^106^ Krychman said that the pretext of management of sexual dysfunctions lies on a three-tier approach of open communication, medical understanding, and education.^107^

In a systematic review and metanalysis by Chow et al., they concluded that with respect to sexual functioning, psychoeducational interventions appeared to have benefits in improving sexual life.^94^ Brotto et al. evaluated a 90-minute mindfulness-based cognitivebehavioral intervention for sexual dysfunction in gynecologic cancer survivors compared with a wait-list control group.

Survivors of endometrial or cervical cancer who self-reported significant and distressing sexual dysfunction (desire and/or sexual arousal) concerns were assigned either to three, 90-minute mindfulness-based cognitive behavior therapy sessions or 2 months of wait-list control before entering the treatment arm. Treatment led to significant improvements in all domains of sexual response, and a trend toward significantly reducing sexual distress.^108^ Other studies have also discussed about the beneficial effects of psychoeducational interventions.^109,110^

### Preventive therapies

A review by Boa and Grénman reported that preventative strategies during surgery should be adopted if possible. These may include limited lymph node dissection to prevent lymphedema by identifying sentinel lymph node, nerve-sparing techniques, and ovary preservation to prevent premature menopause.^21^ The improvements in bladder and bowel function also lead to better sexual function indirectly. Regular sexual activity maintains vaginal health and halts vaginal atrophy.^111^

### Bupropion

Many cervical cancer survivors are being prescribed selective serotonin reuptake inhibitors for depressive symptoms. They have been linked to lower libido and anorgasmia. This may lead to the persistence of depressive symptoms^.111^ Bupropion lacks serotonergic activity. It has noradrenergic and dopaminergic actions resulting in lower incidence of sexual dysfunction.^111^ Bupropion is being studied in a randomized controlled trial in gynecological cancer patients in the doses of 150 and 300 mg to better the sexual desire compared with placebo. The results of this trial are awaited.^112^

## Discussion

This scoping review has discussed the sensitive issue of sexual functioning in cervical cancer survivors. Since cervical cancer is being diagnosed at relatively early stages, a significant number of women experience issues with sexual functioning.^2^ Sexual dysfunction experienced by cervical cancer patients pertains to multiple domains due to the cancer itself and the treatment modalities.^4,27,29–31,42–47^

The younger patients and the ones getting radiotherapy get affected the most.^8,11,13,14,22,23^ Treatment of cervical cancer comprises surgery (radical hysterectomy/fertility sparing surgeries), chemotherapy, and radiotherapy. The patient undergoing surgery experienced an initial decline in sexual function scores, which improved over a period of time, while the effect of radiotherapy persisted for long post-treatment. So, modern methods of radiotherapy are being evaluated.^14,38–41,48–53^

Sexual functioning should be thoroughly evaluated in cervical cancer patients pre- and post-treatment. There are various validated questionnaires available to assess sexual functions of cervical cancer survivors. FSFI is the most widely used with excellent reproducibility of results.^14,40,77^ The effect of cervical cancer diagnosis is not only physical, rather it also involves multiple facets of patients' lives such as social, emotional, and psychological domains. The patients should be supported by their health care providers to maintain healthy relationship with the spouse. The gynecologic oncology surgeons and gynecologic oncology specialist nurse could make the patient and partner comfortable by asking queries and providing counseling.^82,106^

While reviewing therapies, we found that treatment-specific randomized-controlled trials in cervical cancer survivors for sexual dysfunction are lacking. Psychoeducational interventions, HRT, vaginal estrogen creams, lubricants, vaginal dilator therapy, ospemifene, and nerve sparing surgeries have shown some promising effect on sexual dysfunction in cervical cancer survivors.^21,90,100,101,103,104^ Some interventions such as pelvic floor muscle therapy, bupropion, and arousal devices are yet to be proved effective in cervical cancer patients.^79,89,112^ However, we discussed them to open the scope for future researches in the field.

## Conclusions

For too long, researchers have focused on defining the prevalence and types of sexual problems after various cancer treatments. The area that continues to be neglected is the evaluation of effective interventions to prevent or treat cancer-related sexual dysfunction. In particular, mental health and medical specialists need to collaborate to create cost-effective treatment programs. Collaborative intervention with gynecologists, sexologists, radiotherapists, and nursing staff would be beneficial to optimize the sexual wellness of cancer survivors and their spouses. More research and randomized-controlled trials are needed to know the magnitude of problem and its amelioration.

## Abbreviations Used

AC: adenocarcinomas
CARES-SF: Cancer Rehabilitation Evaluation System-Shorter Format
CT: chemotherapy
EORTC: European Organization for Research and Treatment of Cancer
EROS-CTD: EROS-clitoral therapy device
FACT-Cx: Functional Assessment of Cancer Therapy-Cervix
FACT-G: functional assessment of cancer therapy-general
FSD: female sexual dysfunction
FSDS-R: Female Sexual Distress Scale-Revised
FSFI: Female Sexual Function Index
FSS: fertility sparing surgery
HRQoL: health related-quality of life
HRT: hormone replacement therapy
LACC: laparoscopic approach to cervical cancer
MEN-QOL: Menopause-Specific Life Questionnaire
NACT: NeoAdjuvant chemotherapy
QOL: quality of life
RS: radical surgery
RT: radiotherapy
SAQ: Sexual Activity Questionnaire
SCC: squamous cell carcinomas
WHO: World Health Organization
